# Ccr2-64i and Ccr5 Δ32 Polymorphisms in Patients with Late-Onset Alzheimer’s disease; A Study from Iran (Ccr2-64i And Ccr5 Δ32 Polymorphisms in Alzheimer’s disease)

**Published:** 2012

**Authors:** Hamid Reza Khorram Khorshid, Mehdi Manoochehri, Leila Nasehi, Mina Ohadi, Mehdi Rahgozar, Koorosh Kamali

**Affiliations:** 1 *Genetics Research Centre, University of Social Welfare and Rehabilitation Sciences, Tehran, Iran*; 2 *Reproductive Biotechnology Research Centre, Avicenna Research Institute (ACECR), Tehran, Iran *; 3 *Epidemiology and Biostatistics Department, University of Social Welfare and Rehabilitation Sciences, Tehran, Iran*

**Keywords:** Alzheimer’s disease, Genetic Association study, CCR2, CCR5, Inflammation, Iranian population

## Abstract

**Objective(s):**Alzheimer’s disease (AD) is a complex disease with multifactorial etiology. Inflammation has been proven to have an important role in the pathogenesis of AD. Both CCR2 and CCR5 genes expression increase in AD patients comparing to control subjects. CCR5 gene encodes a protein which is a member of the beta chemokine receptors family of integral membrane proteins. *CCR5*-Δ32 is a genetic variant of *CCR5* and is characterized by the presence of a 32-bp deletion in the coding region of the gene, which leads to the expression of a nonfunctional receptor, and the *CCR2*-64I has a change of valine to isoleucine at codon 64, in the first transmembrane domain. It has been proved that both genes have important roles in different stages of inflammation.

**Materials and Methods:**The frequencies of *CCR5*∆32 and *CCR2*-64I variations were determined in 156 AD patients and 161 control subjects using polymerase chain reaction (PCR) and restriction fragment length polymorphism (RFLP) methods, and the results were compared among AD and healthy controls.

**Results** **:S**tatistical analysis showed no significant difference in the distributions of *CCR5*∆32 and *CCR2*-64I between the AD patients and healthy controls (*P*> 0.05). Stratifying the samples by gender, genetic background and presence of ApoEε4 allele showed no significant effect on the distributions of *CCR5*∆32 and *CCR2*-64I (*P*> 0.05).

**Conclusion:**Our study did not show an association between *CCR5*∆32 and *CCR2*-64I variations and AD in the Iranian population. Further confirmatory studies with bigger number of samples are recommended.

## Introduction

As the most common neurodegenerative disorder, Alzheimer’s disease (AD) currently affects 20 to 30 million people worldwide (1). The molecular and cellular mechanisms responsible for the etiology and pathogenesis of AD have not been fully defined (2). However, experimental studies suggest that inflammation plays a fundamental function in the pathogenesis of AD (3); and several community-based studies have linked anti-inflammatory interventions to a lowered risk of developing AD (4). 

Microglias are the mononuclear phagocytes of the brain and their numbers increase in the AD brain, which are clustered in and around Amyloid Beta (Aβ) deposits (5). Evidence for the presence of microglia in senile plaques derives from immunohistochemical studies that examined the brains of AD patients (6). Electron microscopy studies have shown that those microglias are closely opposed to Aβ (7). *In vitro* studies have shown that Aβ directly activates microglia, leading to production of reactive oxygen and nitrogen species, tumor necrosis factor (TNF-α), IL-1 (8, 9) and complement proteins (10). Interaction of microglia with Aβ also leads to secretion of chemokines, such as monocyte chemotactic protein-1 (MCP-1, also known as CCL2). 

Chemokines are a family of proinflammatory cytokines that can stimulate the target-cell-specific directional migration and recruitment of leukocytes to sites of inflammation via interaction with a family of chemokine receptors (11). They and their receptors constitute a large set of proteins, and two subfamilies, CXC and CC chemokines depending on whether they express a CC or CXC amino acid motif in their N-termini, and their receptors, have been identified (12), which coordinate cellular responses to inflammation insult or injury (13). There are growing evidences that chemokines and their receptors are unregulated in AD brain (14) CCR2 and CCR5 are two types of CC receptors, which predominantly express on monocytes surfaces (15). These receptors can bind and signal to different CC chemokines including MCP-1 (CCL2) and RANTES (16, 17).

Hetero-dimeric CCR2 and CCR5 interaction may be implicated in the *in vivo* processes that hamper leukocyte rolling on blood vessels and induce leukocyte parking in tissues during inflammatory responses (18). Indeed, abolishing such accumulation, as occurs in mice deficient in the chemokine receptor CCR2 leads to development of early visible Aβ deposits, specifically around blood vessels, and has been associated with increased mortality in these mice (19). CCL2 is a potent monocyte chemoattractant. Binding of CCL2 to its receptor, CCR2 also stimulates production of reactive oxygen species (20). CCL2 and CCR2 seem to have important functions in the recruitment of mononuclear cells into tissues in both acute and chronic inflammation, and targeted disruption of the CCR2 gene cause decreasing recruitment of monocytes into the peritoneum in a model of acute inflammation (21). Thus, CCR2 controls the recruitment and/or infiltration of mononuclear phagocytes into the brain and CCL2–CCR2 interactions seem to play a key part in recruitment and/or activation of microglia to sites of Aβ deposition in AD. CCR2 deficiency leads to lower microglia accumulation and higher brain β-amyloid levels, indicating that early microglia accumulation promotes Aβ clearance (22). In addition to CCL2, other chemokines and their receptors have been shown to be expressed in Aβ-stimulated monocytes and microglia in AD brain (14). Previous immunohistochemical studies of AD brains have shown that the chemokine receptor CCR5 is present on microglia of both control and AD brains, with increased expression on reactive microglia associated with amyloid deposits in AD suggesting that CCR5 might play a function in the regulation of the brain immune response in AD (23). 

Recent study provides clear evidence that peripheral T cells of AD patients overexpressed MIP-1α, which binds to CCR5 on brain endothelial cells to promote T cells transendothelial migration across the blood–brain barrier (24). In addition, CCR5 is a necessary membrane co-receptor for the binding and entry of human immunodeficiency virus (HIV) into target cells (25). Both *CCR2* and *CCR5* genes are characterized by polymorphisms in their sequence. A single nucleotide polymorphism (SNP) in the *CCR2* gene causes a conservative change of a valine with an isoleucine at codon 64 (*CCR2*-64I), in the first transmembrane domain of the receptor (26) and a 32-bp deletion (*CCR5*Δ32) in the coding region which leads to the expression of a nonfunctional and truncated receptor (27). Previous researches, have reported that these two variations may be involved in AD progression (28- 31). 

Because inflammation may play an important role in progression of Alzheimer’s disease, and *CCR2* and *CCR5* have a primary function in recruitment of leukocytes to inflammatory sites, we hypothesized that these variations might influence the risk of developing AD in our population.

## Materials and Methods

This case control study was conducted in the Genetics Research Centre, University of Social Welfare and Rehabilitation Sciences, Tehran, Iran Inthisstudy,totally156Alzheimer’s patients and 161 healthy controls from Iranian population in several elderly care centres in Tehran city were recruited. The Alzheimer’s disease in patients were diagnosed and confirmed by psychiatrist according to criteria introduced in the DSM-IV and control subjects were selected by the assessment of their medical histories and physical conditions. The participants older than 65 years old were included. An agreement was made prior to their entering the study. The main inclusion criterion for the case group was the diagnosis of AD by DSM-IV criteria; in control group, had the participants have any serious neurologic or psychological disorder, they were excluded from the study. The participants or their families were asked about some personal and baseline information, also ethnicity, job, educational level and gender was considered as co variable (Table 1).

Five ml of peripheral blood samples were collected in tubes containing 200 µl of 0.5 M EDTA; genomic DNA was extracted from peripheral blood using the salting out method, and two pairs of primers were used for amplifying and analyzing the mentioned variations, which are presented in Table 2. The total volume of 25 μl containing 30 ng genomic DNA, 10 pmol of each primer, 1 μl dNTP mix (Fermentas, Life Science), 2.5 μl 10× buffer and 0.5 U of Taq DNA polymerase (Fermentas Life Science, Lithuania) with 1.5 mM MgCl_2_ were mixed in the 0.5 ml Eppendorf microtube for amplification of target sequences. PCRs were performed for 33 cycles, and 95 ˚C pre-denaturation for 4 min followed by denaturation at 94 ˚C for 45 sec, annealing at (60 ˚C for CCR5 and 58 ˚C for CCR2) for 30 sec, extension at 72 ˚C for 40 sec and final extension at 72 ˚C for 5 min.* CCR5*Δ32 genotyping was determined by PCR and 8% polyacrylamid gel electrophoresis (PAGE). PCR for amplifying 220 bp wild type and 188 bp variant type products was carried out (Figure A). For *CCR2*-64I genotyping, PCR-RFLP and 8% polyacrylamid gel electrophoresis (PAGE) were used and *BsaB*I restriction enzyme was performed for 171 bp PCR product digestion. PCR product digestion created 152- and 19-bp fragments for variant type (*CCR2*-V64I) and no digestion, therefore 171 bp intact fragment for wild type (Figure B). We obtained informed consent from participants or their families. 

**Table 1:** Comparison of mean age, gender, job, education level and genetic background between Alzheimer^,^s disease (AD) cases and control subjects

		AD patients (n=156)	Control subjects (n=161)	*P *value
Age		78.55 ± 7.80^a^	77.14 ± 6.95	0.091
Sex (M/F)^b^		63/91	63/99	0.714
Job	Housewife	55.8%	56.2%	0.938
	Own business	23.4%	21.0%	
	Worker	9.2%	8.6%	
	Farmer	3.2%	3.1%	
	Employee	8.4%	11.1%	
Education level	Illiterate	41.6%	43.2%	0.427
	Primary school	29.2%	29.6%	
	Secondary school	16.2%	12.3%	
	Diploma	11.1%	9.3%	
	Academic	1.9%	5.6%	
Genetic background	Fars	61.0%	63.6%	0.490
	Turk	25.3%	25.3%	
	Kurd	3.9%	1.8%	
	Lor	0.7%	2.5%	
	Gilak& Mazani	9.1%	6.8%	

^a^ Mean ± SD; ^b^ Male/Female

**Table 2:** Primer sequences and PCR product sizes

PCR primers	PCR product sizes
*CCR5*Δ32 Variation F: TCT CCC AGG AAT CAT CTT TAC C R: AGC CCT GTG CCT CTT CTT C	Δ32 allele : 188 bp w.t allele: 220 bp
*CCR2*-G/A (V64I) F: TTT GTG GGC AAC ATG ATG G R: GCA CAT TGC ATT CCC AAA G	CCR2 : 171 bp

The data was analyzed using SPSS ver 11.5 (SPSS, Chicago, Ill, USA). Logistic regression analysis was performed to assess the effect of mutant genotype or allele in study groups and related odds ratio (OR) and 95% confidence interval (CI) reported. *P-*values less than 0.05 were considered as significant.

The results were merged with the result of previous study for *APOE *polymorphisms to analyze if the interaction effects of *APOE *with *CCR5* or *CCR2 *are statistically significant.

## Results

The distributions of *CCR5* and *CCR2 *genotype and allele frequencies of each group are summarized in Table 3. There were no statistical differences in *CCR5* and *CCR2 *genotypes and allele frequencies in AD compared to healthy controls (*P*> 0.05). Also there were no significant differences between male and female in both AD patients and health controls, when stratified by gender (*P*> 0.05). No ∆32/∆32 genotype was detected among controls and Alzheimer's patients. 

When we stratified the *CCR5* and *CCR2* results with *ApoEε4* allele for synergic effects, by logistic regression, we could not find any significant differences between combined genotypes for risk of AD (*P*>0.05) (Table4).

**Table 3:** Genotype and allele frequencies of SNPs in the human *CCR2* and CCR5 genes in Alzheimer^,^s disease (AD) patients and controls

	AD patients N=156	Controls N=161	*P*-value	Odds ratio
CCR5 w.t./w.t.	149 (95.5%)	153 (95%)	Reference group	
CCR5 w.t./Δ32	7 (4.5%)	8 (5%)	0.95	1.1 (0.39-3.15)
w.t. allele	305 (97.8%)	314 (97.5%)	0.95	1.1 (0.4-3.1)
Δ32 allele	7 (2.2%)	8 (2.5%)		
CCR2 w.t./w.t.	133 (85.2%)	131(81.4%)	Reference group	
CCR2 w.t./ 64I	21 (13.5%)	28 (17.4%)	0.41	1.35 (0.73-2.5)
CCR2 64I/64I	2 (1.3%)	2 (1.2%)	0.62	0.98 (0.14-7.1)
w.t allele	287 (92%)	290 (90%)	0.48	1.27 (0.73-2.2)
64I allele	25 (8%)	32 (10%)		

**Table 4:** Allele frequencies of *CCR5 *Δ32 and *CCR2*-64I variations in cases and controls based on *APOE *ε4 allele

*APOE *ε4	*CCR5*Δ32	AD patients	Controls	*P*-value
-	-	115 (73.7%)	146 (90.7%)	0.85
-	+	6 (3.8%)	8 (5%)	
+	-	35 (22.4%)	7 (4.3%)	0.50
+	+	1(0.6%)	0	
*ApoE *ε4	*CCR2*-64I	AD patients	Controls	
-	-	102 (65.5%)	129 (80.1%)	0.91
-	+	18 (11.5%)	25 (15.5%)	
+	-	30 (19.2%)	6 (3.7%)	0.69
+	+	6 (3.8%)	1(0.6%)	

**Figure A:**
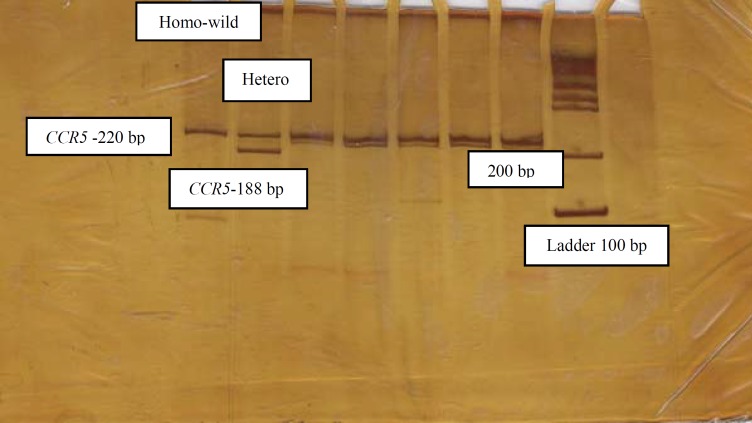
PCR amplification of the human *CCR5* gene. No mutant homozygote variant was found in this study

**Figure B:**
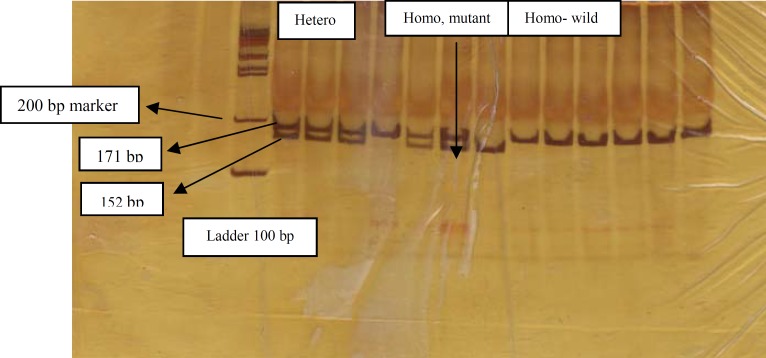
PCR amplification and restriction digestion of *CCR2* by *BsaB*I

## Discussion

In our study performed for the investigation of *CCR5 ∆32* and *CCR2*-64I variations association with the risk of late onset AD, the sample size consisted of 156 Alzheimer’s patients and 161 control subjects. After genotypes and alleles comparison analyses between the two groups (patients and controls) using χ^2^ test, no association between *CCR5 ∆32 *genotypes and AD was found [OR= 1.1 (95% CI= 0.39-3.15)]. In the next step analysis was performed for *CCR5* genotypes and alleles between females and males; that has the same result and no difference in genotypes distribution between Alzheimer’s patients and control subjects by gender stratification was identified (*P*= 0.274 and *P*= 0.280 for males and females, respectively). 

In contrast to other organs, the brain does not show a classical immune response, so it is believed to be immune privileged (32).

Chronic inflammation is assumed to have an important role to disease progression through the production of inflammatory mediators (33). Neuroinflammation is also involved in the pathogenesis of many neurodegenerative disorders (14) and markers of neuroinﬂammation are prominent in numerous CNS disorders including Parkinson’s disease (34), multiple sclerosis (35) and AD. 

There are many evidences indicating the presence of inflammatory reactions during the Alzheimer’s pathogenesis (14). *CCR2* and *CCR5* are chemokine receptors expressed on microglia that mediate accumulation of leukocytes at sites of inflammation. It was suggested that both *CCR2* and *CCR5* expression were increased in AD patients compared to controls; but two studies from Italy (28, 31) and two studies from Spain (29, 30) showed no statistically significant differences between AD and control groups. Mohaddes Ardebili showed significant association for CCR2 but no association for CCR5 in West Northern part of Iran (Eastern Azerbaijan) (36). These chemokine receptors were more known to act as co-receptors for HIV entry into the leukocytes, as *CCR5*∆32 and *CCR2*-V64I have shown to create high resistance against AIDS progression. The well-known polymorphism *CCR5*∆32 that results in a truncated protein is not distributed equally among the world’s population and the north European Caucasians have the highest frequency compared to other parts of the world. As Gharagozloo showed that the frequency of this polymorphism is low in our country (25), we can speculate that finding a higher accumulation of this polymorphism either in AD patients or normal controls can make it either a risk or protective factor amon our population, respectively. Since Hetero-dimeric *CCR2* and *CCR5* interaction may be implicated in the *in vivo* processes that hinder leukocyte rolling on blood vessels and induce leukocyte parking in tissues during inflammatory responses. It was decided to determine whether these polymorphisms have any association with AD onset in our population or not. 

Considering the fact that Iranian population may have a different variations compared to the European population, it should be mentioned that therefore sequencing the genes may result in finding new population specific variations. These new variants may have significant correlations with LOAD comparing to control group. Sample size was low in this study, and further confirmatory studies with bigger number of samples are suggested as well.

## Conclusions

Our study failed to show an association between *CCR5*∆32 and *CCR2*-64I variations and Alzheimer's disease in the Iranian population.
